# Intraoperative Alcohol Withdrawal Syndrome: A Coincidence or Precipitation?

**DOI:** 10.1155/2013/761527

**Published:** 2013-07-10

**Authors:** Asish Subedi, Balkrishna Bhattarai

**Affiliations:** Department of Anaesthesiology and Critical Care, BP Koirala Institute of Health Sciences, Dharan 56700, Nepal

## Abstract

As the prevalence of alcohol dependence is approximately half in surgical patients with an alcohol use disorder, anesthetist often encounters such patients in the perioperative settings. Alcohol withdrawal syndrome (AWS) is one of the most feared complications of alcohol dependence and can be fatal if not managed actively. A 61-year-old man, alcoholic with 50 h of abstinence before surgery, received spinal anesthesia for surgery for femoral neck fracture. To facilitate positioning for spinal anesthesia, fascia iliaca compartmental block with 0.25% bupivacaine (30 mL) was administered 30 min prior to spinal block. Later, in the intraoperative period the patient developed AWS; however, the features were similar to that of local anesthetic toxicity. The case was successfully managed with intravenous midazolam, esmolol, and propofol infusion. Due to similarity of clinical features of AWS and mild local anesthetic toxicity, an anesthetist should be in a position to differentiate the condition promptly and manage it aggressively.

## 1. Introduction

Alcohol dependence (AD) is high in patients with an alcohol use disorder (AUD) presenting for surgery [1]. Therefore, encountering such patients in anesthetic practice is not unlikely. However, 1%–24% of surgical patients with a history of AUD are missed during routine clinical assessment [2, 3]. Dependent patients show higher morbidity and have more adverse events such as infection or cardiopulmonary complication. Alcohol withdrawal syndrome (AWS) is one of the most feared complications of AD, and if untreated the mortality is as high as 15% [4]. Although AWS in perioperative setting is observed more commonly in the postoperative period, we report a case of AWS that developed in the intraoperative period and review the possible factors for its precipitation.

## 2. Case Description

A 61-year-old man weighing 50 kg was scheduled for open reduction and internal fixation of traumatic intertrochanteric fracture left femur under regional anesthesia. He had a history of chronic alcoholism with 50 h of abstinence before surgery. Physical examination was unremarkable. Laboratory findings revealed mild elevation of liver enzymes. With overnight fasting, the patient received aspiration prophylaxis, prophylactic intravenous multivitamins, and oral lorazepam 2 mg at bedtime and 2 h before surgery. On arrival to the block room, intravenous line was secured and standard monitoring was applied. Left fascia iliaca compartmental block (FICB) with bupivacaine 0.25% (30 mL) was administered via landmark technique to facilitate positioning for spinal anesthesia. After 30 min of the block, patient received spinal anesthesia with hyperbaric bupivacaine 0.5% (2.5 mL) and fentanyl 20 micrograms (0.2 mL) in sitting position.

Following spinal anesthesia, patient was shifted to the operating table, and midazolam 2 mg IV was given. After 20 min of surgical incision, patient became agitated and started talking irrelevantly. He was confused, disoriented, and developed tachycardia, hypertension, and tremors. Airway was maintained, and supplemental oxygen at 8 lit/min was started through face mask. Boluses of intravenous midazolam up to 10 mg were administered. For hypertension and tachycardia, esmolol infusion was initiated and titrated accordingly. With persistent agitation and difficulty to restrain, propofol infusion was started and maintained at 50 mcg/kg/min. Once the vitals were stable and the patient became sedated, the surgical procedure was continued.

At the end of the surgery, patient was shifted to intensive care unit (ICU) while continuing the propofol infusion. A psychiatric consultation in the ICU confirmed the diagnosis of AWS. In the ICU, both propofol and lorazepam infusion were initiated. Over the next 50 h, propofol infusion was gradually reduced and stopped. Once the Clinical Institute Withdrawal Assessment of Alcohol Scale, Revised (CIWA-Ar) score reached <10, patient was kept on oral lorazepam. The patient was shifted to the ward on the 4th postoperative day and discharged from the hospital on the 12th day.

## 3. Discussion

In AD surgical patients, events such as hypotension, hypoxia, and uncontrolled pain in the perioperative period may precipitate them to AWS. In our patient, the syndrome manifested intraoperatively under regional anesthesia, and an interesting observation we want to highlight from this case report is whether there was any association between local anesthetic toxicity and manifestation of AWS.

In AD patients, the incidence of AWS has been observed to be higher in surgical and trauma patients than the other patients group [5, 6]. Abstinence from drinking following hospital admission in these patients put them at risk of AWS. Our patient did not show any features suggestive of AWS during and after performing FICB. However, later in the operating room he manifested the features of AWS. Apart from alcohol cessation itself being a risk factor for AWS in our patient, surgical stress could have augmented the syndrome further, since the literature suggests the stress of surgery as one of its precipitating factors [6].

Withdrawal is characterized by brain hyperexcitability due to reduced GABA activity and increased glutaminergic action [7]. Also, increased noradrenergic activity during withdrawal leads to sympathetic overdrive [8]. Similar mechanism of depression of central cortical inhibitory pathways, leaving the excitatory pathways to function unopposed, is postulated with the initial central toxic effects of local anesthetics. Further, CNS mediated sympathetic nervous system activation can occur at lower toxic LA concentration leading to hypertension and tachycardia [9]. Hence, there is a possibility of further exaggeration of AWS in our patient from activated excitatory pathways and sympathetic surge of mild LA toxicity.

The likely explanation of LA toxicity is that our patient with relatively old age had preexisting ethanol-induced hepatic dysfunction, and thereby, impairment of bupivacaine metabolism might have played a role in its toxicity. Although patients with mild hepatic impairment may have single-shot blocks with normal doses, great care and due consideration have been recommended in these patients when administering amide LAs especially with continuous infusion [9].

With increased sympathetic activity and altered mental status, there are several causes that can mimic features of AWS [4]. Since there is a similarity between the clinical features of lower toxic level of LA and AWS, a differential diagnosis of LA toxicity should always be kept in mind if the amide LA is used especially with landmark techniques. Due to unavailability of USG, we have been performing the peripheral nerve block safely with landmark techniques. However, had it been done under USG guidance, the location would have been more precise and smaller volume of LA could have been used, thereby enhancing the safety margin.

In our case, the patient was identified as AD only a day before surgery and was missed at the early stage. Although we initiated the AWS prophylaxis after its identification, it did not confer any protection against manifestation of AWS. It has been reported that 25% of the AD patients still manifest AWS despite use of preoperative preventive strategies [10]. The risk may be higher in emergency cases or semiemergency cases like ours, where there is time constrain for a preventive strategy to be more effective.

## 4. Conclusion

Since there is a resemblance between the clinical presentation of AWS and local anesthetic toxicity, an anesthetist must always have a high index of suspicion of LA toxicity as a precipitating factor or differential diagnosis of AWS, especially if amide LA-based regional anesthesia is used.
